# Development and internal validation of a screening tool for chronic prostatitis (S-CP)

**DOI:** 10.1007/s00345-023-04574-x

**Published:** 2023-09-15

**Authors:** Yoichiro Tohi, Yasukazu Hijikata, Mikio Sugimoto, Hideya Kuroda, Mineo Takei, Takakazu Matsuki, Tsukasa Kamitani, Yoshiyuki Kakehi, Shunichi Fukuhara, Yosuke Yamamoto

**Affiliations:** 1 Department of Urology, Faculty of Medicine, Kagawa University, 1750-1, Ikenobe, Miki-Cho, Kita-Gun, Kagawa, 761-0701 Japan; 2 Department of Healthcare Epidemiology, School of Public Health in the Graduate School of Medicine, Kyoto University, Kyoto, Japan; 3 Section of Clinical Epidemiology, Department of Community Medicine, Graduate School of Medicine, Kyoto University, Kyoto, Japan; 4 Kuroda Urology Clinic, Osaka, Japan; 5 Department of Urology, Harasanshin General Hospital, Fukuoka, Japan; 6 Matsuki Urology Clinic, Kagawa, Japan; 7 Section of Education for Clinical Research, Kyoto University Hospital, Kyoto, Japan; 8 Department of Health Policy and Management, Johns Hopkins Bloomberg School of Public Health, Baltimore, USA; 9 Shirakawa STAR for General Medicine, Fukushima Medical University, Fukushima, Japan

**Keywords:** Prostatitis, Surveys and questionnaires, Prevalence, Undiagnosed diseases, Clinical decision rules

## Abstract

**Purpose:**

We developed a simple self-checkable screening tool for chronic prostatitis (S-CP) and internally validated it to encourage men (in the general population) with possible chronic prostatitis to consult urologists.

**Methods:**

The expert panel proposed the S-CP, which comprises three domains: Area of pain or discomfort (6 components), accompanying Symptom (6 components), and Trigger for symptom flares (4 components). We employed logistic regression to predict chronic prostatitis prevalence with the S-CP. We evaluated the predictive performance using data from a representative national survey of Japanese men aged 20 to 84. We calculated the optimism-adjusted area under the curve using bootstrapping. We assessed sensitivity/specificity, likelihood ratio, and predictive value for each cutoff of the S-CP.

**Results:**

Data were collected for 5,010 men—71 (1.4%) had a chronic prostatitis diagnosis. The apparent and adjusted area under the curve for the S-CP was 0.765 [95% confidence interval (CI) 0.702, 0.829] and 0.761 (0.696, 0.819), respectively. When the cutoff was two of the three domains being positive, sensitivity and specificity were 62.0% (95% CI 49.7, 73.2) and 85.4% (95% CI 84.4, 86.4), respectively. The positive/negative likelihood ratios were 4.2 (95% CI 3.5, 5.2) and 0.45 (95% CI 0.33, 0.60), respectively. The positive/negative predictive values were 5.7 (95% CI 4.2, 7.6) and 99.4 (95% CI 99.1, 99.6), respectively.

**Conclusion:**

The reasonable predictive performance of the S-CP indicated that patients (in the general population) with chronic prostatitis were screened as a first step. Further research would develop another tool for diagnostic support in actual clinical settings.

**Supplementary Information:**

The online version contains supplementary material available at 10.1007/s00345-023-04574-x.

## Introduction

Chronic prostatitis/chronic pelvic pain syndrome (CP), grouped as category III in the National Institutes of Health (NIH) classification, is a common disease in men, with an estimated prevalence of 1.8–9.7% of the male population [1, 2]. CP significantly impairs the quality of life (QOL) and is widely distributed among older adults and young people, who play a central role in economic activities [3]. Therefore, CP is a disease that significantly impacts society [4].

Patients with CP present with various symptoms, primarily pelvic pain, discomfort, and dysuria. However, CP diagnosis is complex. No definitive diagnostic method for CP has been established, and no consensus has been reached regarding its diagnosis. The gold standard is a comprehensive diagnosis by an experienced urologist involving the exclusion of other urological disorders [5]. Consequently, physicians in general practice and urology clinics, who are mainly the primary care providers for patients with CP [6], have been facing challenges in diagnosing and managing this condition and reported confusion and frustration [7, 8].

Moreover, there is concern that a significant number of patients with CP may lack proper diagnosis and treatment. In fact, in our previously published work [3], a survey of the general population depicted that the percentage of participants who reported CP-like symptoms without CP diagnosis was more than double the percentage of those who had been diagnosed with CP at medical institutions (3.7% vs. 1.4%, respectively). Notably, both groups had a significantly impaired QOL. Therefore, it is crucial to provide more patients with CP with the opportunity to receive appropriate treatment by developing simple, self-checkable screening tools to encourage individuals (in the general population) affected by CP to consult a urologist.

In the past 20 years, there have been over 2,000 reports on CP. There has been much discussion on the molecular mechanisms of this intractable disease and how to treat it [9]. Nevertheless, reports to improve diagnostic opportunities have been limited. Several studies have been conducted to estimate the prevalence of CP in the general population using the NIH-Chronic Prostatitis Symptom Index (CPSI) [10]. However, the NIH-CPSI is a tool primarily designed to measure symptom severity in patients with CP, not for screening for actual CP [11].

Therefore, we developed and internally validated the screening tool for CP (S-CP), a self-checkable clinical prediction model (CPM) for CP extraction in the general population, to recommend medical consultation to those who may be affected by CP.

## Methods

### Study design and population

This study used data from a questionnaire survey distributed electronically via the Nippon Research Center, an independent research company, from January 5 through March 4, 2021. The participants were men aged 20 to 84 years at the time of the survey. Those who could not read or write Japanese were excluded from this study. Data from those who answered all the questions were included in the analysis. The survey was designed to represent the general population of Japan based on quota sampling from the national census in 2015 using quota sampling [12] and continued until the target of 5,000 men was reached at each stratum. The survey details and the target population’s characteristics are described elsewhere [3]. This study was conducted in accordance with the World Medical Association’s Declaration of Helsinki [13]. It was approved by the Institutional Review Board of Kagawa University (approval number: 2020-064) and the Institute for Health Outcomes and Process Evaluation Research (approval number: 202001). All participants provided written informed consent. This study followed the Transparent Reporting of a Multivariable Prediction Model for Individual Prognosis or Diagnosis (TRIPOD) reporting guidelines [14].

### Data collection

The target condition for prediction was the prevalence of CP, defined as having a history of CP diagnosis at a medical facility. Participants who answered “yes” to the question, “Have you ever been diagnosed with CP?” were assigned to the CP group. Additionally, we extracted data on the participants’ background characteristics (e.g., age, comorbidity, and lifestyle) and the predictors for CP prevalence that comprised the CPM described below.

### Screening tool for CP (S-CP)

We formed an expert panel comprising two urologists working at a university hospital, one urologist working at a general hospital, two urologists working at a clinic, and three epidemiologists to determine the domains and items that constitute a simple CPM for predicting the prevalence of CP, which was named S-CP. Each domain and its items were then extracted with reference to the RAND/UCLA appropriateness method [15]. Multiple panel meetings were held until a consensus was reached. First, we determined the three key domains in the screening for CP. We then discussed the items which comprised each domain and created an item pool consisting of 23 items. Finally, we selected the 16 critical items that comprised the three domains. We agreed to consider a domain as positive if the participants presented at least one of the items of that domain. Besides, we decided to predict CP prevalence using three dichotomous variables representing each domain. Domain 3 was determined if either domain 1 or domain 2 was positive (i.e., if domains 1 and 2 were negative, domain 3 would also be negative). The different domains and their items are listed below.

**Domain 1. “Area” of pain or discomfort (6 items):** “area between rectum and testicles (perineum), testicles, urethra of the penis, tip of the penis (not related to urination), below the waist in the pubic or bladder area, groin area.”

**Domain 2. Accompanying “Symptom” (6 items):** “pain or burning during urination, pain or discomfort during or after sexual climax (ejaculation), hematogenous semen or hematuria, discoloration of semen, premature ejaculation, erectile dysfunction.”

**Domain 3. “Trigger” for symptom flares (4 items):** “cold, sitting or driving, stimulant intake or alcohol consumption, lack of sleep or stress.”

### Statistical analyses

The distribution of background characteristics for all participants was first described. Then, participants with CP were compared to those without CP. Continuous variables were summarized using the median and interquartile range. Dichotomous variables were summarized using numbers and percentages. P-values for differences in characteristics were calculated from the Wilcoxon rank sum test for continuous variables and the Fisher’s exact test for dichotomous variables.

Second, a logistic regression analysis was performed to explain the prevalence of CP with the three variables: “Area” of pain or discomfort, accompanying “Symptom,” and “Trigger” for symptom flares. Each variable’s intercept and regression coefficients were calculated to develop a temporal S-CP. We calculated the area under the curve (AUC) and 95% confidence interval (CI) of the temporal S-CP to estimate the ability for apparent discrimination. Optimism-adjusted AUC and bootstrap shrinkage factor were also calculated using bootstrapping (200 times) [16]. The temporal S-CP was then uniformly shrunk using the bootstrap shrinkage factor [17], and the shrinkage model was referred to as the final version of the S-CP. The risk for each stratum of the final S-CP is illustrated as a risk table. In addition, the sensitivity, specificity, positive/negative likelihood ratio, and positive/negative predictive value were calculated when the cutoff was defined as all the three domains of “Area,” “Symptom,” and “Trigger” being positive. The analysis was also performed when the cutoff was defined as positive for two of the three domains. To assess calibration, we compared the estimated risk of CP using the final S-CP with the observed proportion for each stratum. All data were analyzed using Stata version 17.0 (StataCorp LLC, College Station, TX, USA).

## Results

The survey request was distributed to 19,450 men, and 6,671 (34.3%) indicated a willingness to participate. Of these, 5,010 (75.1%) provided complete responses, and their data were analyzed. In total, 71 (1.4%) had a history of CP diagnosis. The distribution of background characteristics for all participants and participants grouped with and without CP is shown in Table 1. Substantial differences were observed between the CP and no-CP groups in the distribution of most items except for the “Groin area” and “Erectile dysfunction” that are part of the S-CP. Differences in the distribution of comorbidity (cardiovascular disease, diabetes, depression, male infertility) were also observed, but there was no marked difference in lifestyle.

**Table 1:** Distribution of participant background

	All participants	CP	No CP	*P*-value
	5010	71 (1.4)	4939	
Age	51 (38–65)	55 (39–71)	51 (38–65)	0.13
Comorbidity				
Cardiovascular disease	197 (3.9)	19 (27)	178 (3.6)	< 0.001
Diabetes	560 (11)	24 (34)	536 (11)	< 0.001
Depression	294 (5.9)	12 (17)	282 (5.7)	< 0.001
Male infertility	31 (0.6)	5 (7.0)	26 (0.5)	< 0.001
Lifestyle				
Smoking habit	1246 (25)	23 (32)	1223 (25)	0.17
Drinking habit	2757 (55)	42 (59)	2715 (55)	0.55
Sexual activity (per month)	3 (0–10)	2 (1–5)	3 (0–10)	0.25
Area	708 (14)*	40 (56)	668 (14)	< 0.001
Area between rectum and testicles (perineum)	206 (4.1)	16 (23)	190 (3.8)	< 0.001
Testicles	132 (2.6)	8 (11)	124 (2.5)	< 0.001
Urethra of the penis	137 (2.7)	17 (24)	120 (2.4)	< 0.001
Tip of the penis (not related to urination)	95 (1.9)	8 (11)	87 (1.8)	< 0.001
Below the waist, in the pubic or bladder area	147 (2.9)	9 (13)	138 (2.8)	< 0.001
Groin area	280 (5.6)	7 (9.9)	273 (5.5)	0.12
Symptom	1560 (31)*	50 (70)	1510 (31)	< 0.001
Pain or burning during urination	116 (2.3)	15 (21)	101 (2.0)	< 0.001
Pain or discomfort during or after sexual climax (ejaculation)	156 (3.1)	14 (20)	142 (2.9)	< 0.001
Hematogenous semen or hematuria	83 (1.7)	7 (9.9)	76 (1.5)	< 0.001
Discoloration of semen	99 (2.0)	10 (14)	89 (1.8)	< 0.001
Premature ejaculation	254 (5.1)	11 (16)	243 (4.9)	< 0.001
Erectile dysfunction	1312 (26)	27 (38)	1285 (26)	0.029
Trigger**	550 (11)*	32 (45)	518 (11)	< 0.001
Cold	206 (4.1)	22 (31)	184 (3.7)	< 0.001
Sitting or driving	166 (3.3)	21 (30)	145 (2.9)	< 0.001
Stimulant intake or alcohol consumption	89 (1.8)	14 (20)	75 (1.5)	< 0.001
Lack of sleep or stress	386 (7.7)	23 (32)	363 (7.3)	< 0.001

Data are presented as number (%) or median (interquartile range). *CP* chronic prostatitis/chronic pelvic pain syndrome. For continuous variables, the p values are from the Wilcoxson rank sum test, and for dichotomous variables, they are from the χ2 test. *Percentage of those with at least one of the items. **Evaluated if one of the items of “Area” or “Symptom” was applicable

### The screening tool for CP (S-CP)

The intercept for the temporal S-CP generated via logistic regression analysis was − 5.410, and the regression coefficients for the “Area,” “Symptom,” and “Trigger” were 1.470, 0.989, and 0.778, respectively. The apparent AUC for the temporal S-CP was 0.765 (95% CI 0.702–0.829). The optimism-adjusted AUC calculated by applying bootstrapping was 0.761 (95% CI 0.696–0.819), and the bootstrap shrinkage factor was 0.982. The linear predictor for the final S-CP with uniform shrinkage had an intercept of − 5.377 and regression coefficients of 1.444, 0.972, and 0.764 for the “Area,” “Symptom,” and “Trigger,” respectively.

The risk of CP prevalence for each stratum using the final S-CP is shown in Table 2. When the cutoff was defined as all the three domains of “Area,” “Symptom,” and “Trigger” being positive, the sensitivity and the specificity were 35.2% (95% CI 24.2– 47.5) and 96.1% (95% CI 95.5–96.6), respectively. The positive and negative likelihood ratios were 9.0 (95% CI 6.4–12.6) and 0.67 (95% CI 0.57–0.80). The positive and negative predictive values were 11.4 (95% CI 7.5–16.4) and 99.0 (95% CI 98.7–99.3), respectively. When the cutoff was defined as two of these three domains being positive, the sensitivity and the specificity were 62.0% (95% CI 49.7–73.2) and 85.4% (95% CI 84.4–86.4), respectively. The positive and negative likelihood ratios were 4.2 (95% CI 3.5, 5.2) and 0.45 (95% CI 0.33–0.60), respectively. The positive and negative predictive values were 5.7 (95% CI 4.2–7.6) and 99.4 (95% CI 99.1–99.6), respectively. The final version of the S-CP and the risk table is available in the supplementary material.

**Table 2:** Risk table of CP prevalence calculated using the screening tool for CP (S-CP)

	Area (+)		Area (−)	
	Symptom (+)	Symptom (−)	Symptom ( +)	Symptom (−)
Trigger (+)	10	4	2.6	N/A
Trigger (−)	4.9	1.9	1.2	0.5

Area (6 items): area between rectum and testicles (perineum), testicles, urethra of the penis, tip of the penis (not related to urination), below the waist in pubic or bladder area, and groin area

Symptom (6 items): pain or burning during urination, pain or discomfort during or after sexual climax (ejaculation), hematogenous semen or hematuria, discoloration of semen, premature ejaculation, and erectile dysfunction

Trigger (4 items): coldness, sitting or driving, stimulant intake or alcohol consumption, and lack of sleep or stress

**Fig. 1:**
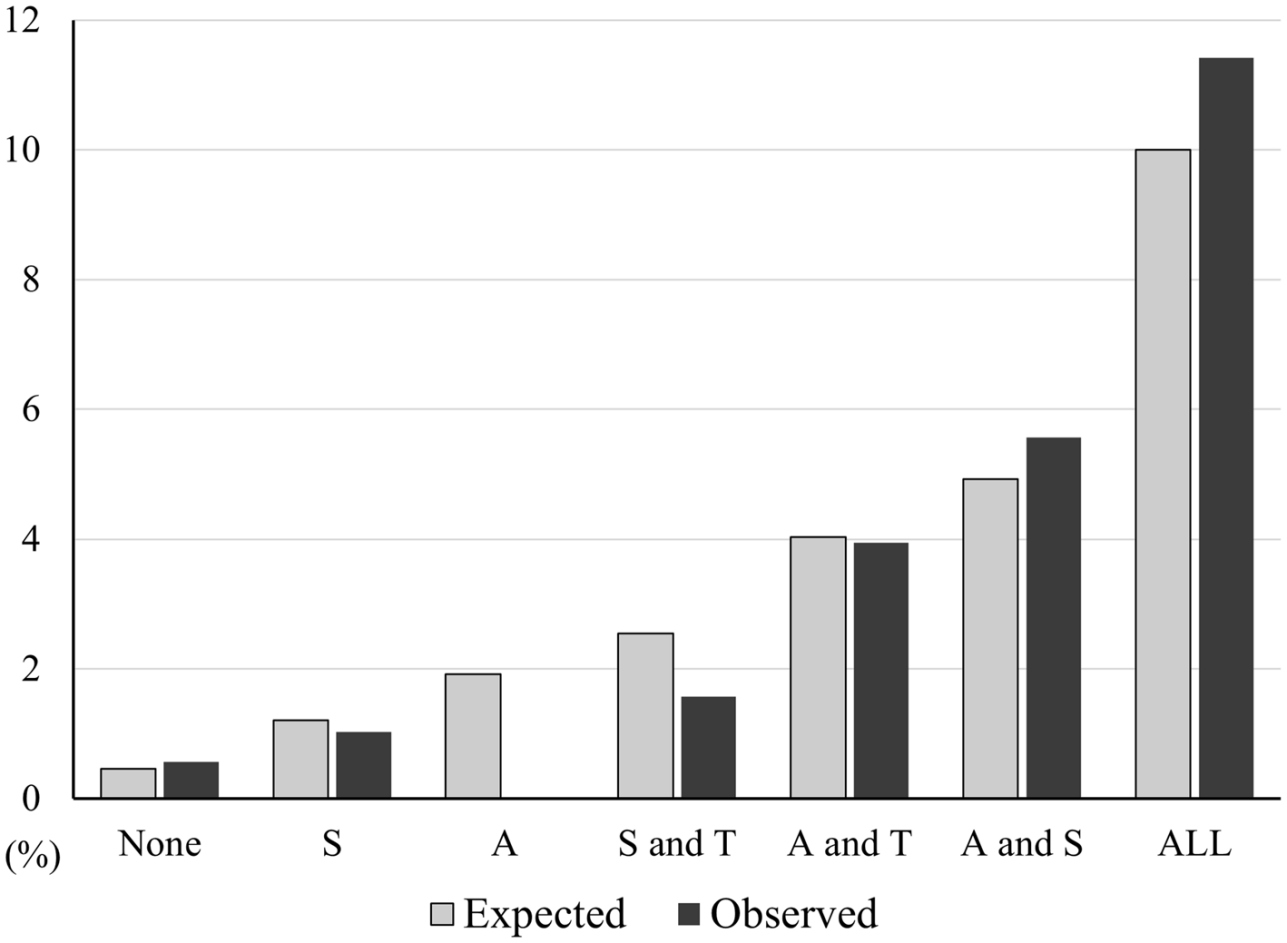
Prevalence of CP expected by screening tool for CP (S-CP) and observed value. The vertical axis indicates the expected and observed risk of CP prevalence (%). *CP* Chronic prostatitis/chronic pelvic pain, *A* Area of pain or discomfort, *S* accompanying Symptom, *T* Trigger for symptom flares

Figure 1 shows the expected risk of CP prevalence and the observed CP prevalence in each stratum using the final S-CP. Although no CP prevalence was observed in the strata to which only “Area” was applicable, the expected risk and observed prevalence were generally well-calibrated. The number of participants and patients with CP in each stratum are shown in Supplementary Table 1.

## Discussion

In the present study, we developed and internally validated the S-CP, a simple screening tool for CP prevalence in the general population. The discrimination power of CP prevalence using the S-CP is relatively high, with an optimism-adjusted AUC of 0.761 (95% CI 0.696–0.819), indicating that this tool is helpful for the initial screening of patients with CP from the general population. Recommendations for urologist evaluation in high-risk populations identified using the S-CP may facilitate appropriate diagnosis and treatment in patients who may have CP.

CP is not a monogenic disease but rather a heterogeneous syndrome with many aspects, including urinary factors, psychosocial factors, organ specificity, infection, neurogenic/neuropathic factors, and tenderness of skeletal muscles [18–20]. CP diagnosis requires sufficient clinical experience and is often problematic, resulting in a significant number of undiagnosed patients in the general population [3, 21]. Therefore, there is an urgent need to provide possible patients with CP with the opportunity to receive appropriate diagnosis and treatment. To achieve this goal, we could first develop a CPM that enables easy self-assessment of the risk of CP prevalence in the general population and subsequently recommends those at high risk to undergo medical examinations with a urologist. The S-CP is a simple and self-checkable tool composed solely of information collected via a questionnaire survey. Considering that CP is a highly multifaceted syndrome, the AUC of 0.761 (95% CI 0.696–0.819) seemed to be an adequate discriminative performance. Therefore, the S-CP is expected to significantly contribute to improving CP care.

For the clinical implementation of the S-CP, consideration of how to recommend medical examination with a urologist is essential. Specifically, it is necessary to decide whether the cutoff for the S-CP should be positive in all three domains or two of the three domains. The negative predictive value is clinically essential since the S-CP is used to screen for possible patients with CP in the general population by self-check. The negative predictive values for the cutoffs of “all the three” and “two of the three” were high [99.0 (95% CI 98.7–99.3) and 99.4 (95% CI 99.1–99.6), respectively]. However, when the cutoff was set to “all the three,” the number of false negatives increased, reaching 46/71 patients.

Therefore, we suggest that “two of the three” should be adopted as the cutoff (27/71 false negatives). When the cutoff was set to “two of the three,” the positive predictive value was only 5.7 (95% CI 4.2–7.6), indicating a high frequency of false positives. In this case, people would be burdened with unnecessary anxiety and the cost of visiting a medical facility. However, we expect many false positives to be diagnosed with other urologic conditions that present similar symptoms to CP. Given the benefit of the S-CP in providing possible patients with CP or those with CP-like conditions to see a urologist, the degree of false positives could be acceptable.

There were several limitations to this study. First, there is a possibility of misclassification for CP prevalence. The determination of CP prevalence was based on self-reports of diagnostic history. Thus, patients diagnosed with acute prostatitis or other similar conditions could have responded that they had a diagnosis of CP. There is no certainty as to the accuracy of the CP diagnosis at each clinic. Moreover, possible patients with CP would be misclassified as having no CP within this study population. However, the prevalence of diagnosed CP cases in the study population (1.4%) was generally consistent with those in previous studies of diagnosed CP cases [2, 11]. Second, the sample size was limited. Although the study population was relatively large (over 5,000), only 71 events were available for analysis because of the low prevalence of CP. More precise prediction might be possible if risk estimation could reflect the associations and interactions among the 16 items of the S-CP. However, this would require a considerable number of patients with CP to be sampled from the general population, which is impractical. Third, external validation is yet to be performed. Although internal validation has been performed, this study is a development study of the S-CP, and external validation is desired for its clinical implementation. In addition, the target population of this study was limited to Japanese individuals, and it is desirable to validate the diagnostic performance of the S-CP in other countries. Finally, the S-CP could not be used for diagnostic support in clinical settings as it was developed for use in the general population. The next step in providing possible patients with CP with the opportunity to receive appropriate diagnosis and treatment is to improve the accuracy of CP diagnosis in actual clinical settings, which could be achieved with another CPM to support CP diagnosis. The prevalence of comorbidities differs between the CP and no-CP groups, likewise in the domains of the S-CP (Table 1). Based on the findings of this study and additional clinically available information, such as physical examination of the abdomen, genitalia, perineum, and prostate [22], we believe that it is possible to develop a CPM to support the diagnosis of CP in actual clinical settings.

In conclusion, we developed and internally validated the S-CP, a simple screening tool for CP prevalence in the general population. Recommendations to see a urologist in high-risk populations identified using the S-CP may enable possible patients with CP to receive timely diagnosis and treatment. Further research is warranted to develop another CPM for diagnostic support in clinical settings.

### Supplementary Information

Below is the link to the electronic supplementary material.

Supplementary file1 (DOCX 81 KB)

Supplementary file2 (DOCX 16 KB)

## Data Availability

The datasets created and analyzed in this study are not publicly available. Requests for data can be addressed to the corresponding author (MS).
