# Supplementary material for: Development and internal validation of a screening tool for chronic prostatitis (S-CP)
Source: World J Urol. 2023 Sep 15;41(10):2759–65. doi: 10.1007/s00345-023-04574-x (PMC10582131; doi:10.1007/s00345-023-04574-x)
Supplement: Supplementary file 1 — Supplementary file1 (DOCX 81 KB) [file 345_2023_4574_MOESM1_ESM.docx]

**Supplementary Material**

Article Title: Development and Internal Validation of a Screening Tool for Chronic Prostatitis (S-CP)

Journal name: World Journal of Urology

Author names: Yoichiro Tohi, Yasukazu Hijikata, Mikio Sugimoto, Hideya Kuroda, Mineo Takei,
Takakazu Matsuki, Tsukasa Kamitani, Yoshiyuki Kakehi, Shunichi Fukuhara,
Yosuke Yamamoto

Corresponding author: Mikio Sugimoto
 Department of Urology, Faculty of Medicine, Kagawa University
 [sugimoto.mikio@kagawa-u.ac.jp](file:///C:\Users\Yasukazu%20Hijikata\Desktop\CP2本目投稿フォルダ\投稿用資料\BJUI\PCPD\YOOHI_20_3_06062023-0811620\WJU\Minor_Revision\sugimoto.mikio@kagawa-u.ac.jp)

【**Screening Tool for Chronic Prostatitis (S-CP)】**

**Q1. “Area” of pain or discomfort**

In the past year, have you experienced pain or discomfort in any of the following areas?
Please answer Yes if at least one of these applies to you, and No if none applies to you.

**
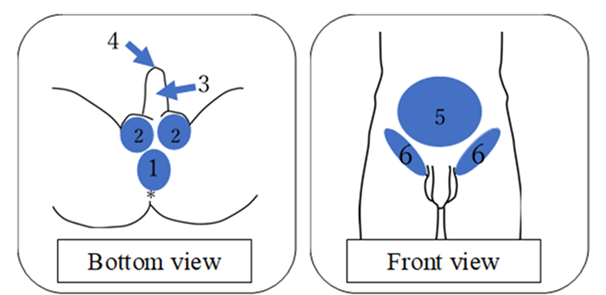
**

| 1. Area between rectum and testicles (perineum) 2. Testicles 3. Urethra of the penis 4. Tip of the penis (not related to urination) 5. Below the waist in the pubic or bladder area 6. Groin area |
| --- |

**Q2. Accompanying “Symptom”**

In the past year, have you experienced any of the following?
Please answer Yes if at least one of these applies to you, and No if none applies to you.

| 1. Pain or burning during urination 2. Pain or discomfort during or after sexual climax (ejaculation) 3. Hematogenous semen or hematuria 4. Discoloration of semen 5. Premature ejaculation 6. Erectile dysfunction |
| --- |

**Q3 “Trigger” for symptom flares**

If you answered Yes to either or both **Q1 Area** and **Q2 Symptom**, we ask you this question.
Did those symptoms worsen under the following situations?
Please answer Yes if at least one of these applies to you, and No if none applies to you.　

| 1. Cold 2. Sitting or driving 3. Stimulant intake or alcohol consumption 4. Lack of sleep or stress |
| --- |

**【Interpretation of the results】**

Risk table of Chronic Prostatitis prevalence calculated using the screening tool for CP (S-CP)

|  | Q1 Area (Yes) |  | Q1 Area (No) |  |
| --- | --- | --- | --- | --- |
|  | Q2 Symptom (Yes) | Q2 Symptom (No) | Q2 Symptom (Yes) | Q2 Symptom (No) |
| Q3 Trigger (Yes) | 10 | 4 | 2.6 | N/A |
| Q3 Trigger (No) | 4.9 | 1.9 | 1.2 | 0.5 |

The number in the cell that applies to you indicates the likelihood that you have chronic prostatitis. We recommend that anyone who answers Yes to two or more of Q1, Q2, and Q3 consult a urologist.
