# Supplementary material for: Development and internal validation of a screening tool for chronic prostatitis (S-CP)
Source: World J Urol. 2023 Sep 15;41(10):2759–65. doi: 10.1007/s00345-023-04574-x (PMC10582131; doi:10.1007/s00345-023-04574-x)
Supplement: Supplementary file 2 — Supplementary file2 (DOCX 16 KB) [file 345_2023_4574_MOESM2_ESM.docx]

**Supplementary Table 1.**

Article Title: Development and Internal Validation of a Screening Tool for Chronic Prostatitis (S-CP)

Journal name: World Journal of Urology

Author names: Yoichiro Tohi, Yasukazu Hijikata, Mikio Sugimoto, Hideya Kuroda,
Mineo Takei, Takakazu Matsuki, Tsukasa Kamitani, Yoshiyuki Kakehi,
Shunichi Fukuhara, Yosuke Yamamoto

Corresponding author: Mikio Sugimoto
 Department of Urology, Faculty of Medicine, Kagawa University
 <sugimoto.mikio@kagawa-u.ac.jp>

The number of participants and patients with CP in each stratum

| Stratum | Expected prevalence  of CP (%) | Number of participants | Number  of CP (%) |
| --- | --- | --- | --- |
| None | 0.5 | 3177 | 18 (0.6) |
| S | 1.2 | 870 | 9 (1.0) |
| A | 1.9 | 197 | 0 (0) |
| S and T | 2.6 | 255 | 4 (1.6) |
| A and T | 4.0 | 76 | 3 (4.0) |
| A and S | 4.9 | 216 | 12 (5.6) |
| All | 10 | 219 | 25 (11) |
| CP, Chronic prostatitis/chronic pelvic pain; A, Area of pain or discomfort; S, accompanying Symptom; T, Trigger for symptom flares | | | |
